# Functional Outcomes in Failed Osteosynthesis of Proximal Femoral Fractures Managed by Total Hip Replacement Using the Modified Hardinge Approach: An Ambispective Cohort Study

**DOI:** 10.7759/cureus.89671

**Published:** 2025-08-09

**Authors:** Narendra S Kushwaha, Utkarsh Upadhyay, Arpit Singh, Mohd Yoosuf, Sujeet K Chaudhary, Ashish Kumar

**Affiliations:** 1 Department of Orthopaedic Surgery, King George's Medical University, Lucknow, IND

**Keywords:** failed osteosynthesis, harris hip score (hhs), modified hardinge approach, proximal femoral fracture, total hip arthroplasty

## Abstract

Introduction

Proximal femoral fractures are a major cause of disability, particularly in aging populations, with an increasing incidence. Although osteosynthesis remains the first-line treatment, failures are common due to various complications. Total hip arthroplasty (THA) is the preferred salvage procedure in such cases, despite its technical challenges. This study aims to evaluate functional outcomes following THA in patients with failed proximal femoral fracture fixation.

Materials and methods

This ambispective cohort study was conducted from 2022 to 2023 at King George’s Medical University. Adult patients who underwent rescue THA for failed proximal femoral fixation were included. Clinical and radiological evaluations were performed preoperatively. All surgeries were performed under spinal anesthesia using a modified Hardinge approach. Postoperative functional outcomes were assessed using the Harris Hip Score (HHS) at follow-ups. Data were analyzed using descriptive statistics and appropriate inferential tests.

Results

A total of 39 patients (26 males, 13 females; mean age = 57.5 ± 9.9 years) were included, with most aged between 50 and 70 years. Fracture types included femoral neck (n = 21), intertrochanteric (n = 16), and subtrochanteric (n = 2). Common prior fixation methods included proximal femoral nail (33.3%), cannulated cancellous screws (25.6%), and dynamic hip screws (20.5%). The causes of failure included avascular necrosis (30.8%), backout (20.5%), non-union (17.9%), cut-out (12.8%), and secondary arthritis (17.9%). Patients underwent uncemented (41%), cemented (38.5%), or hybrid (20.5%) THA. Mean Harris Hip Scores improved significantly from preoperative (61.2 ± 10.3) to 6 weeks (74.1 ± 2.6), 1 month (82.5 ± 1.7), 6 months (88.0 ± 2.0), and 1 year (92.5 ± 1.1) postoperatively (p < .0001).

Conclusion

Total hip arthroplasty provides favorable functional outcomes in the management of failed osteosynthesis of proximal femoral fractures when guided by thorough preoperative planning and surgical expertise. The primary aim remains the restoration of hip biomechanics and facilitation of early mobilization. However, limitations such as small sample size and short follow-up duration warrant further multicentric studies for validating long-term outcomes.

## Introduction

Proximal femoral fractures, including femoral neck, intertrochanteric, and subtrochanteric fractures, constitute a substantial burden on the healthcare system, especially in aging populations. In India, the annual incidence exceeds 120 cases per 100,000 individuals over 50 years, with projections indicating a significant rise due to demographic shifts and increased life expectancy [1-3].

Although osteosynthesis is the standard initial intervention, failure rates remain notable due to complications such as implant cut-out, avascular necrosis, non-union, and secondary osteoarthritis [4-6]. Failed fixation not only results in persistent pain and disability but also necessitates revision procedures to restore mobility and improve quality of life.

Total hip arthroplasty (THA) serves as the preferred salvage option in such cases. However, revision THA following failed osteosynthesis is technically more challenging than primary THA due to altered anatomy, poor bone stock, soft tissue compromise, and increased risk of infection or dislocation [7-9]. Despite these concerns, THA has shown promising functional outcomes when performed with meticulous planning and surgical expertise.

There is limited literature from the Indian subcontinent evaluating functional outcomes of rescue THA performed via the modified Hardinge approach in cases of failed osteosynthesis. This study aims to bridge this gap by assessing the clinical outcomes, specifically Harris Hip Score improvement, and surgical experiences in patients managed with THA after failed internal fixation of proximal femoral fractures.

## Materials and methods

Study design and setting

An ambispective cohort study with both prospective and retrospective follow-up was conducted over a 30-month period, spanning from January 2022 to December 2024, at a high-volume tertiary care academic center in North India. King George's University Institutional Ethics Committee's approval was obtained prior to initiation of the study (reference code: XXVI-PGTSC-IIA/P20).

Patient selection

This study included patients who underwent total hip arthroplasty (THA) as a salvage procedure following failed internal fixation of proximal femoral fractures. Failure of osteosynthesis was defined by the presence of complications such as implant cut-out, backout, non-union, avascular necrosis, malalignment, or development of secondary arthritis. Patients with active implant-related infections are excluded.

**Figure 1:**
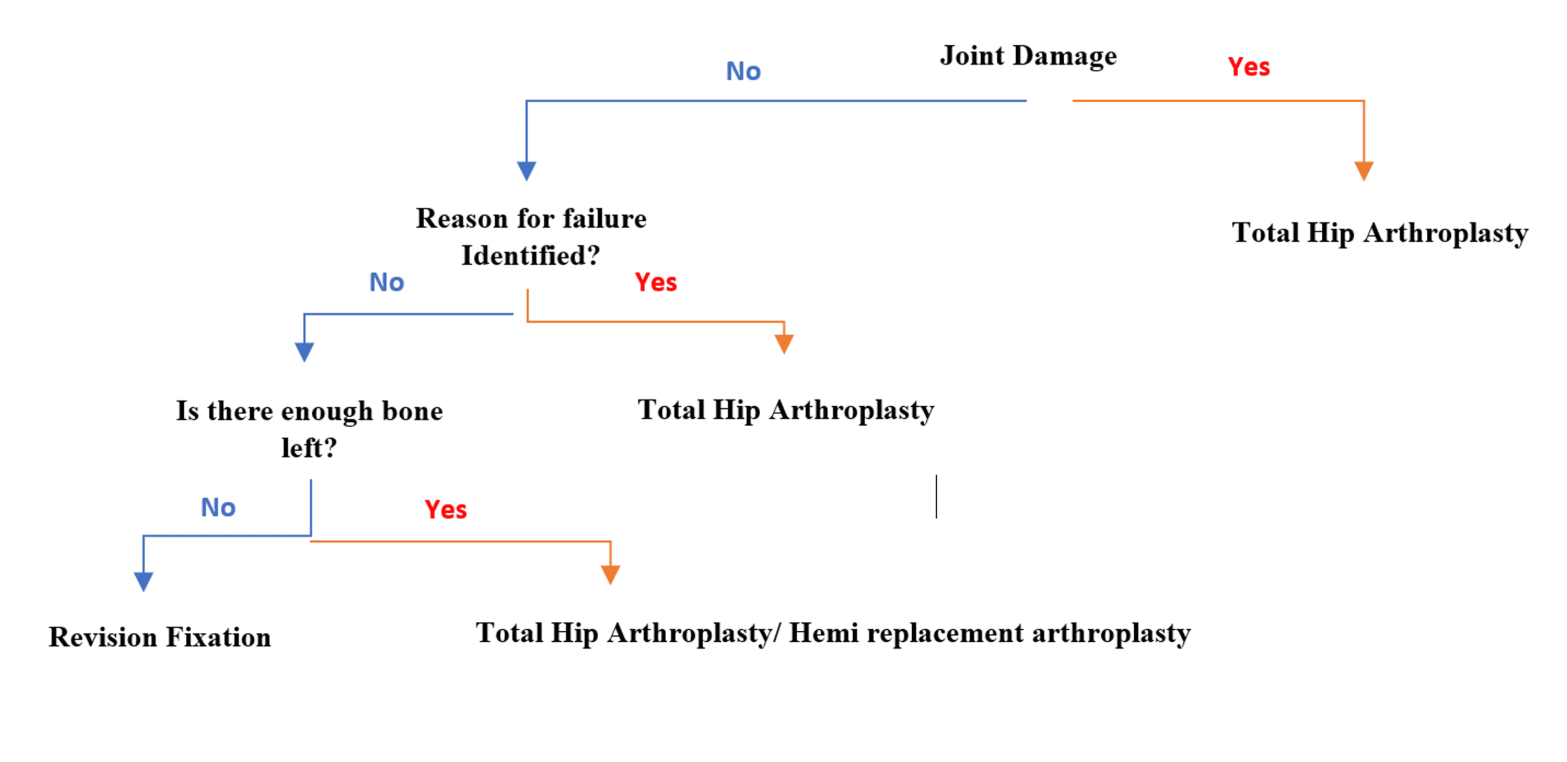
Flow chart of approach in failed osteosynthesis in proximal femur fracture.

Patients with hip pain with an implant in situ were selected in the outpatient clinic of the orthopaedics department at King George's Hospital, Lucknow. X-rays of the hip area were done. Further, patients were planned according to the flow chart given in Figure 1.

Preoperative evaluation

All included patients underwent a comprehensive preoperative assessment comprising clinical examination, limb length discrepancy evaluation, and radiographic imaging (anteroposterior and lateral views of the pelvis and affected femur) and Harris Hip Score. Routine blood investigations were performed to assess general fitness for surgery and screen for infection. These included complete blood count, liver and renal function tests, fasting and post-prandial blood glucose, ESR, CRP, and viral serologies (HIV, hepatitis B surface antigen (HBsAg), hepatitis C virus (HCV)). Preoperative planning with digital templating was conducted to assess implant sizing and alignment. All patients planned for surgery were informed about the study and surgery, and they provided written informed consent.

Surgical procedure

All surgeries were performed by experienced arthroplasty surgeons using a standardized technique. Under spinal anesthesia, patients were positioned in the lateral decubitus position and approached through a modified Hardinge (direct lateral) approach [10]. After exposing the hip joint, previous fixation devices were carefully removed.

A femoral neck osteotomy was performed according to preoperative templating. The acetabulum was cleared of all remaining cartilage and sclerotic bone until subchondral bleeding was evident. In patients with protrusio acetabuli, morselized femoral head autograft was used to restore the acetabular medial wall. After sequential reaming, an appropriately sized acetabular cup was implanted.

The femoral canal was prepared using hand reamers and broaches. Femoral stems were selected based on the preoperative plan and inserted accordingly. Trial components were used to assess hip stability and leg length. Definitive prosthetic components were implanted, ensuring proper anteversion and alignment. The joint was reduced, and the stability was confirmed through a range of motion testing. The wound was closed in layers over suction drainage, and a sterile dressing was applied.

**Figure 2:**
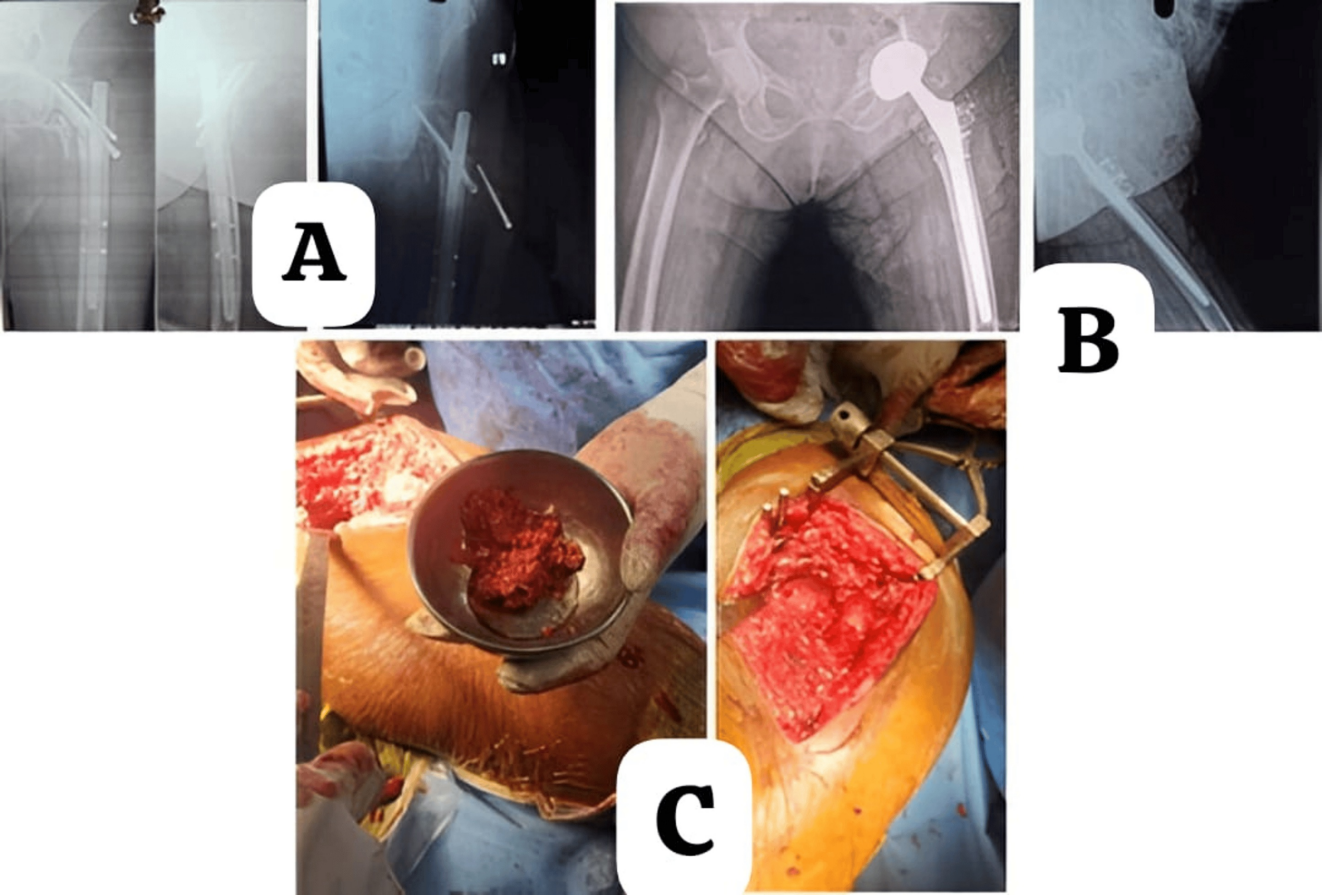
Case of a 69-year-old female with a left intertrochanteric femur fracture and failed osteosynthesis performed at another hospital. Managed with implant and femoral head removal, followed by total hip arthroplasty via the Modified Hardinge approach using autologous bone grafting in the acetabulum. (A) Preoperative X-ray demonstrating screw back-out. (B) Postoperative X-ray following total hip replacement. (C) Impaction grafting of the acetabulum using an autologous graft prepared from the femoral head with a bone mill and impacted into the acetabular floor to address a medial wall defect.

**Figure 3:**
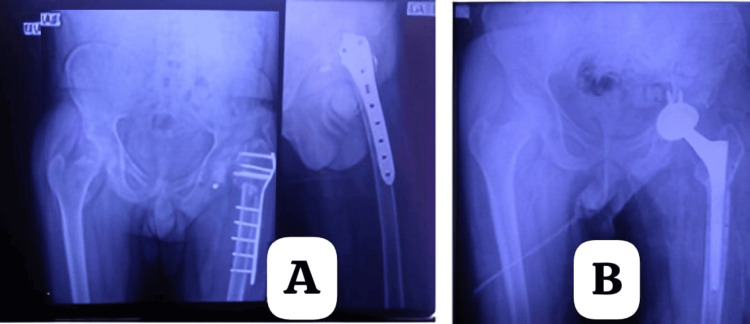
A 55-year-old male with a left intertrochanteric femur fracture and ipsilateral femoral neck fracture, with failed implant in situ and secondary osteoarthritis, managed with single-stage long-stem uncemented total hip replacement. (A) Preoperative X-ray showing a distal femur locking compression plate and avascular necrosis of the femoral head with arthritis. (B) Postoperative X-ray following total hip arthroplasty.

Case scenario images are given in Figure 2 and Figure 3.

Postoperative protocol

Postoperative care included prophylactic intravenous antibiotics (piperacillin tazobactam injection of 4.5 g intravenous with 0.5 g intravenous injection the night before surgery, followed by piperacillin tazobactam injection 4.5 g intravenous 30 min before surgery) and thromboembolism prevention as per institutional protocols. Physiotherapy was initiated on postoperative day one. In cemented THA cases, full weight-bearing was allowed by day three. In uncemented THA, toe-touch weight-bearing was started by day three, as most of the surgeries were revision surgeries, and these need time for osteointegration, progressing to full weight-bearing by 12 weeks.

Follow-up and outcome assessment

Patients were followed clinically and radiologically at 6 weeks, 3 months, 6 months, and 12 months. Functional outcome was assessed using the Harris Hip Score (HHS) at each visit. Radiographs were evaluated for implant position, signs of loosening, and other complications.

Statistical analysis

Data were recorded and managed using Microsoft Excel (Microsoft Corporation, Redmond, USA). Statistical analysis was performed using SPSS version 24.0 (IBM Corp., Armonk, USA). Continuous variables were summarized as mean ± standard deviation (SD), while categorical variables were expressed as counts and percentages. Preoperative and postoperative HHS scores were compared using paired t-tests and one-way ANOVA as appropriate. A p-value of <0.05 was considered statistically significant.

## Results

A total of 39 patients were included in the final analysis, comprising 26 males and 13 females, with a mean age of 57.54 ± 9.86 years (range: 41-76 years). The majority of patients (n=29, 74.4%) were within the age group of 50 to 70 years, which aligns with the demographic commonly affected by osteoporotic and high-energy trauma-related proximal femoral fractures.

The types of fractures initially managed by osteosynthesis included femoral neck fractures in 21 patients (53.8%), intertrochanteric fractures in 16 patients (41.0%), and subtrochanteric fractures in two patients (5.1%). These fractures had initially been treated with a variety of internal fixation techniques; however, subsequent mechanical and biological failures necessitated revision with total hip arthroplasty (THA). The most frequently used implant prior to THA was the proximal femoral nail (PFN), utilized in 13 patients (33.3%), followed by cannulated cancellous screws in 10 cases (25.6%), and dynamic hip screws (DHS) in eight cases (20.5%). Other forms of osteosynthesis included K-wire or pin fixation (n=6, 15.4%) and distal femur plating (n=2, 5.1%), as shown in Table 1.

**Table 1:** Distribution of index osteosynthesis procedures

Preoperative Implant	Number of Patients (n=39)	Percentage (%)
Proximal Femoral Nail (PFN)	13	33.3
Cannulated Cancellous Screws	10	25.6
Dynamic Hip Screw (DHS)	8	20.5
K-wire/Pin Fixation	6	15.4
Distal Femur Plate	2	5.1

The reasons for the failure of the initial osteosynthesis were multifactorial. Avascular necrosis (AVN) of the femoral head was identified in eight patients (20.5%). Mechanical complications were also prominent, with backout of the implant in 12 patients (30.8%) and cut-out in five patients (12.8%). Non-union was observed in seven cases (17.9%), and an equal number of patients developed secondary osteoarthritis (n=7, 17.9%) as a long-term complication. These data are summarized in Table 2.

**Table 2:** Causes of failure of index osteosynthesis

Cause of Failure	Number of Patients	Percentage (%)
Avascular Necrosis (AVN)	8	20.5
Back-Out of Implant	12	30.8
Non-union	7	17.9
Secondary Osteoarthritis	7	17.9
Implant Cut-Out	5	12.8

Following failure of osteosynthesis, all patients were managed with total hip arthroplasty. Among these, uncemented THA was the most commonly employed approach, performed in 16 patients (41.0%). Cemented THA (both femur and acetabulum are cemented) was carried out in 15 patients (38.5%), and the remaining eight patients (20.5%) underwent hybrid THA, wherein a combination of cemented and uncemented components was used depending on bone quality and intraoperative findings.

The functional outcomes were assessed using the Harris Hip Score (HHS), which showed a statistically and clinically significant improvement at each follow-up interval. The mean preoperative HHS was 61.23 ± 10.26, indicating moderate to severe functional impairment at baseline. This improved to 74.13 ± 2.60 at 6 weeks, 82.46 ± 1.73 at 3 months, and 87.97 ± 1.98 at 6 months. At the final one-year follow-up, the mean HHS had increased to 92.49 ± 1.14, demonstrating excellent restoration of function and pain relief. The progression in HHS values across the follow-up period was found to be highly significant (p < 0.0001) using one-way ANOVA. These findings are detailed in Table 3.

**Table 3:** Functional outcome based on the Harris Hip Score (HHS)

Time Point	Mean HHS ± SD	Statistical Significance
Preoperative	61.23 ± 10.26	
6 Weeks Postoperative	74.13 ± 2.60	
3 Months Postoperative	82.46 ± 1.73	
6 Months Postoperative	87.97 ± 1.98	
12 Months Postoperative	92.49 ± 1.14	p < 0.0001 (ANOVA)

Throughout the follow-up period, no major postoperative complications such as dislocation, deep infection, or periprosthetic fracture were documented. However, it is important to note that this study was limited to a follow-up duration of one year, and long-term complications could not be evaluated within the scope of the current study. Intraoperative challenges, such as removal of well-fixed hardware and management of bone defects, were successfully addressed using surgical strategies such as autografting from the femoral head and the use of long-stem implants where necessary.

## Discussion

The present study evaluated the functional outcomes of total hip arthroplasty (THA) as a salvage procedure in cases of failed osteosynthesis of proximal femoral fractures. Our findings indicate a significant improvement in Harris Hip Score (HHS) across all postoperative time points, with patients demonstrating excellent functional recovery and return to ambulation within a year of surgery. These results support the growing consensus that THA provides a reliable and durable solution in complex cases where internal fixation has failed.

Proximal femoral fractures, particularly those involving the femoral neck and intertrochanteric region, are increasingly common in the elderly population due to rising life expectancy and the prevalence of osteoporosis [11,12]. While internal fixation remains the first-line treatment in most cases, failure rates are not insignificant and have been reported to range between 10% and 34%, depending on fracture type, patient age, and comorbidities [13,14]. In our study, the most common causes of fixation failure were avascular necrosis, implant backout, and non-union. These complications are well-documented in the literature and reflect the mechanical and biological challenges associated with fracture healing in osteoporotic bone [15,16].

The functional outcomes observed in this study are consistent with those reported in prior investigations. Nambiar et al. [17] observed a similar trajectory of HHS improvement, reporting a mean score of 60.9 at baseline and over 90 at 1 year post-THA. Neruganti et al. [18] also reported excellent results in their prospective study of rescue THA for failed proximal femoral fixation, emphasizing the importance of surgical experience and proper preoperative planning. A unique feature of our study was the use of the modified Hardinge approach, which offers several advantages in the setting of revision hip surgery [19]. Unlike the posterior approach, which necessitates sacrifice of the short external rotators and may predispose to postoperative dislocation, the lateral approach enables preservation of posterior structures while still providing adequate exposure for component placement.

The distribution of THA types in our study (uncemented in 41.0%, cemented in 38.5%, and hybrid in 20.5%) reflects a pragmatic approach to implant selection based on intraoperative assessment of bone quality [20]. While uncemented implants are increasingly favored in younger patients with adequate bone stock, cemented or hybrid constructs remain valuable in patients with osteoporosis or bone loss. Importantly, we did not encounter any cases of early implant loosening or subsidence, further supporting the appropriateness of our implant selection protocol.

Studies by Rastogi et al. and others [21,22] have emphasized the value of the modified Hardinge technique in enhancing joint stability and reducing the risk of dislocation, which was reflected in our series by the absence of dislocation events during the 1-year follow-up. In our study, the mean HHS improved from 61.23 preoperatively to 92.49 at 1 year, with all patients achieving functional independence by the end of the follow-up period. This level of improvement compares favorably with the work of Bidolegui et al. and D’Arrigo et al., who also reported significant pain relief and restoration of function following salvage THA [23,24].

Despite the encouraging results, this study is not without limitations. The sample size was relatively small, and the follow-up duration was limited to one year. Although early functional outcomes were excellent, complications such as implant wear, late infection, and periprosthetic fracture typically manifest over longer periods and could not be assessed. Moreover, while the surgeries were performed at a high-volume center by experienced surgeons, the generalizability of these findings to lower-resource settings may be limited. We also did not include a comparative cohort of patients managed by hemiarthroplasty or revision fixation, which could have provided additional insight into relative efficacy.

Nevertheless, the study adds meaningful evidence to the growing body of literature advocating for THA as the preferred salvage procedure following failed proximal femoral fixation. Our results affirm that, when performed with careful preoperative planning and intraoperative precision, THA restores hip function effectively and allows patients to regain mobility with minimal complications.

## Conclusions

Total hip arthroplasty (THA) is an effective salvage option for failed osteosynthesis of proximal femoral fractures, offering notable improvements in pain, mobility, and function. This ambispective cohort study showed significant gains in Harris Hip Scores up to 1 year postoperatively. The modified Hardinge approach, tailored implant selection, and thorough preoperative planning contributed to favorable outcomes with minimal early complications.

Despite challenges from prior implants and altered anatomy, THA can reliably restore biomechanics and enable early recovery when performed by experienced surgeons. Limitations include the single-center design, small sample size, and short follow-up. Larger, multicenter studies with long-term outcomes are needed to confirm these findings.
